# How Do Thermophiles Organize Their Genomes?

**DOI:** 10.1264/jsme2.ME23087

**Published:** 2024-06-06

**Authors:** Naomichi Takemata

**Affiliations:** 1 Department of Synthetic Chemistry and Biological Chemistry, Graduate School of Engineering, Kyoto University, Kyoto 615–8510, Japan

**Keywords:** genome organization, thermophilic archaea, reverse gyrase, NAP, SMC

## Abstract

All cells must maintain the structural and functional integrity of the genome under a wide range of environments. High temperatures pose a formidable challenge to cells by denaturing the DNA double helix, causing chemical damage to DNA, and increasing the random thermal motion of chromosomes. Thermophiles, predominantly classified as bacteria or archaea, exhibit an exceptional capacity to mitigate these detrimental effects and prosper under extreme thermal conditions, with some species tolerating temperatures higher than 100°C. Their genomes are mainly characterized by the presence of reverse gyrase, a unique topoisomerase that introduces positive supercoils into DNA. This enzyme has been suggested to maintain the genome integrity of thermophiles by limiting DNA melting and mediating DNA repair. Previous studies provided significant insights into the mechanisms by which NAPs, histones, SMC superfamily proteins, and polyamines affect the 3D genomes of thermophiles across different scales. Here, I discuss current knowledge of the genome organization in thermophiles and pertinent research questions for future investigations.

All organisms must compact and store genomes within the limited confines of micrometer-scaled cellular environments, while also executing a number of DNA-templated processes in a meticulously regulated manner. To fulfill these complex requirements, genomes have evolved intricate spatial organization at diverse scales (Misteli, 2020; Takemata and Bell, 2020; Lioy *et al.*, 2021). In eukaryotes, genomic DNA is packaged into nucleosomes, each of which comprises ~147 bp of DNA wrapped around a histone octamer. Nucleosomal DNA is further organized into higher-order structures, including loops and Topologically Associating Domains (TADs). These structures are largely formed by Structural Maintenance of Chromosomes (SMC) complexes, which are major and widely conserved components of chromosomes. SMC proteins play additional roles in higher-order genome organization, such as holding sister chromatids together and compacting chromosomes for their‍ ‍efficient segregation into daughter cells (Yatskevich *et‍ ‍al.*, 2019). The higher-order architectural landscape of multicellular eukaryotes further exhibits differentiation into transcriptionally active (A-type) and inactive (B-type) compartments. In bacteria, genomic DNA is locally compacted by a number of Nucleoid-Associated Proteins (NAPs) and in turn folded into larger-scale structures termed Chromosomal Interaction Domains (CIDs). CIDs are formed by active transcription at their boundaries. Current evidence posits that bacterial SMC complexes do not participate in the formation of CIDs; instead, these proteins promote chromosome segregation (Niki *et al.*, 1991; Britton *et al.*, 1998; Le *et al.*, 2013; Lioy *et al.*, 2018). DNA topology is another important parameter defining the 3D genome architecture, and prokaryotes and eukaryotes both possess multiple DNA topoisomerases that maintain the proper topological state of genomic DNA (Chen *et al.*, 2013).

Although often overlooked, the spatial and functional organization of genomes is affected by extracellular environments. Environmental factors, such as ionic strength, nutrient availability, pH, and radiation, affect the biophysical properties of DNA directly or indirectly (Ward, 1988; Reynolds *et al.*, 1996; Baumann *et al.*, 1997). Above all, high temperatures have a profound impact on genome organization by melting the DNA duplex, inducing chemical changes in DNA, and increasing the random thermal motion of genomes. These changes may exert many detrimental effects, such as DNA breaks, uncontrolled promoter melting, interference with directed chromosome movement for genome segregation, and hindrances in the search for homologous regions during DNA repair (Lindahl, 1993; Marguet and Forterre, 1994).

In consideration of the harmful impact of high temperatures, it is noteworthy that many microbial species have been isolated from high-temperature environments. Most of these thermophiles are classified under the domains Bacteria and Archaea, with the latter domain being particularly rich in microbes that thrive in hyperthermal environments (>80°C). A growing number of available genome sequences have recently revealed a positive correlation between growth temperature and genomic GC content within some phylogenetic clades. However, a high GC content is not an obligatory requirement for thermophiles, as has been recognized for a long time (Galtier and Lobry, 1997; Hu *et al.*, 2022). Instead, thermophiles (particularly those with an optimal growth temperature higher than 65°C) are most typically characterized by the presence of reverse gyrase, a unique topoisomerase that is considered to protect genomes at high temperatures (Forterre, 2002; Catchpole and Forterre, 2019). Other elements, such as NAPs and polyamines, have also been implicated in enhancing the thermostability of DNA in thermophiles (Sandman *et al.*, 1990; McAfee *et al.*, 1995; Higashibata *et al.*, 1999; Terui *et al.*, 2005; Guo *et al.*, 2008; Hardy and Martin, 2008).

Despite the long-standing interest in thermophiles and their ability to prosper in extreme temperatures, the mechanisms by which they sculpt and preserve the primary, secondary, and tertiary structures of genomes have not yet been elucidated. Furthermore, how these mechanisms allow thermophiles to withstand high temperatures remains largely unknown. Here, I provide an overview of the current state of knowledge regarding the genome organization in thermophiles, with an emphasis on thermophilic archaea as exemplary models. This review delves into four critical areas: the‍ ‍role of reverse gyrase, the mediation of local genome folding by NAPs, the formation of higher-order genome structures, and the role of polyamines in altering local and larger-scale DNA conformations. Crucial questions to be addressed in future work are also discussed.

## Roles of reverse gyrase for the genome integrity of thermophilic archaea

**Fig. 1.**
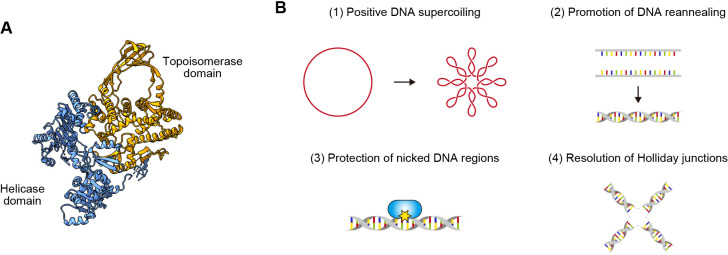
Structure and function of reverse gyrase. (A) Structure of reverse gyrase from *Archaeoglobus fulgidus* (PDB: 1GKU) (Rodríguez and Stock, 2002). (B) Molecular functions of reverse gyrase on DNA.

Distinct from other topoisomerases, reverse gyrase uses energy from ATP to introduce positive DNA supercoiling. This enzyme is composed of an ATP-dependent helicase domain and a type IA topoisomerase domain (Fig. 1A) (Lulchev and Klostermeier, 2014). Reverse gyrase was first identified in 1984 from a cell extract of the extremely thermophilic archaeon *Sulfolobus acidocaldarius* (Kikuchi and Asai, 1984). Two years later, the genome of *Sulfolobus shibatae* virus (SSV1) was shown to be positively supercoiled inside *Sulfolobus* cells (Nadal *et al.*, 1986). In 2002, a comparative genomics study found that the occurrence of reverse gyrase is limited to prokaryotes with an optimal growth temperature higher than 65°C (Forterre, 2002). These cumulative findings led to the widely accepted idea that reverse gyrase prevents the thermal denaturation of DNA by introducing positive DNA supercoiling.

Despite the well-known potential role of reverse gyrase described above, there has been a debate concerning whether and how the positive DNA supercoiling activity of reverse gyrase contributes to cell viability at high temperatures. In members of *Sulfolobus*, which have two reverse gyrase paralogs (referred to as TopR1 and TopR2), heat shock increases positive supercoils in plasmid DNA. This topological alteration coincides with the augmented activity of TopR1 upon the temperature up-shift (López-García and Forterre, 1999; Couturier *et al.*, 2020). This response suggests that the reverse gyrase-dependent formation of positive DNA supercoils is physiologically relevant in thermally challenging environments. However, cross-species ana­lyses using a number of thermophilic archaeal plasmids revealed marked topological variability, spanning from positively supercoiled to even negatively supercoiled (Charbonnier and Forterre, 1994; López-García and Forterre, 1997; López-García *et al.*, 2000). Moreover, some thermophiles possess both reverse gyrase and gyrase, a type IIA topoisomerase that introduces negative DNA supercoiling (López-García *et al.*, 2000). The gyrase gene from the thermophilic bacterium *Thermotoga maritima* was recently overexpressed in the hyperthermophilic archaeon *Thermococcus kodakarensis*. While this overexpression led to the strong negative supercoiling of plasmid DNA, the *T. kodakarensis* strain did not show a clear growth defect (Villain *et al.*, 2021). These studies bring into question the physiological importance of positive DNA supercoiling in the context of high-temperature survival.

Reverse gyrase may play versatile roles in the survival of thermophilic archaea, extending beyond its involvement in maintaining DNA in a positively supercoiled state (Fig. 1B). *In vitro* experiments demonstrated that reverse gyrases from *Archaeoglobus fulgidus* and *Pyrobaculum calidifontis* can promote DNA renaturation, irrespective of topological connections between two DNA strands (Hsieh and Plank, 2006; Jamroze *et al.*, 2014). Another *in vitro* study revealed that *A. fulgidus* reverse gyrase prevents the heat-induced breakage of double-stranded DNA in an ATP-independent manner by coating nicked regions (Kampmann and Stock, 2004). Reverse gyrases from *Sulfolobus solfataricus* (now belonging to the new genus *Saccharolobus* [Sakai and Kurosawa, 2018]) and *P. calidifontis* have the ability to destabilize synthetic Holliday junctions *in vitro* (Valenti *et al.*, 2010; Jamroze *et al.*, 2014). Biochemical studies further demonstrated that reverse gyrase interacts with the translesion DNA polymerase Dpo4 to inhibit its activity in *S. solfataricus* (Valenti *et al.*, 2009). These properties highlight the potential *in vivo* role of reverse gyrase in mitigating or repairing DNA damage arising from thermodenaturation. Experimental support for this hypothesis is provided by a number of *in vivo* observations. For example, reverse gyrase is mobilized to chromosomal DNA in *S. solfataricus* after ultraviolet irradiation (Napoli *et al.*, 2004). In addition, the CRISPR-mediated depletion of TopR1 in *Sulfolobus islandicus* accelerates DNA degradation induced by a DNA alkylation agent (Han *et al.*, 2017).

Despite the controversy surrounding the precise intracellular function of reverse gyrase, genetic studies established that this enzyme is important for the fitness of thermophiles. Thermophiles in the phylum Euryarchaeota possess a single reverse gyrase, and its loss causes growth impairments in the hyperthermophilic euryarchaea *T. kodakarensis* and *Pyrococcus furiosus* (Atomi *et al.*, 2004; Lipscomb *et al.*, 2017). The severity of these impairments correlates with environmental temperature. In both species, growth inhibition is complete at approximately 95°C, which is higher than the optimal growth temperature of wild-type *T. kodakarensis* (85°C), but lower than that of wild-type *P. furiosus* (100°C). In the REY14A strain of the crenarchaeon *S. islandicus*, efforts to individually delete *topR1* and *topR2* proved to be futile (Zhang *et al.*, 2013). In contrast, a more recent study used another *S. islandicus* strain, M.16.4, and succeeded in constructing single deletion mutants of *topR1* and *topR2*. In this case, the loss of *topR2* induced more severe growth retardation than that of *topR1* at an optimal growth temperature (Zhang *et al.*, 2018). Therefore, although reverse gyrase is not essential for all thermophilic archaea, its presence is critical for their survival across a broad spectrum of high temperatures.

## NAPs revisited: How do they organize DNA and contribute to thermophilic life?

NAPs constitute a diverse and prevalent class of DNA-binding proteins in prokaryotic organisms (Peeters *et al.*, 2015; Lioy *et al.*, 2021). They interact with DNA in various manners, including bending, wrapping, coating, and bridging, with low sequence specificity (Dillon and Dorman, 2010). Major archaeal NAPs include homologs of histones, which are pivotal constituents of eukaryotic chromatin (Laursen *et al.*, 2021).

*In vitro* studies revealed that a number of NAPs from mesophilic and thermophilic prokaryotes increase the melting temperature of DNA by up to 40°C (Rouvière-Yaniv *et al.*, 1977; Stein and Searcy, 1978; Sandman *et al.*, 1990; McAfee *et al.*, 1995; Higashibata *et al.*, 1999; Guo *et al.*, 2008; Hardy and Martin, 2008). These findings raise the‍ ‍possibility that NAPs fulfill a specific function in safeguarding genomic DNA against denaturation. Hocher *et al.* recently showed that the relative abundance of NAPs significantly varies among a wide range of archaeal species (from <0.03% to >5% of total protein). They also reported a correlation between overall NAP levels and the growth temperature of these organisms. This led to the hypothesis that thermophilic archaea may employ elevated levels of NAPs for chromatinization, thereby fortifying their genomes against heat-induced denaturation (Hocher *et al.*, 2022). The total abundance of NAPs, rather than (or as well as) the presence of specific NAPs, may be key to thermal adaptation.

Hocher *et al.* (2022) also raised the fascinating possibility that high levels of chromatinization in thermophilic archaea are evolutionarily linked to eukaryotic chromatin, in which a large proportion of genomic DNA is wrapped around histone octamers. Archaeal histone abundance correlates with growth temperature, similar to those of other NAPs. Among a broad range of archaeal species, only thermophilic archaea appear to exhibit sufficient histone abundance for the formation of long nucleosomal arrays (Hocher *et al.*, 2022). In contrast, a previous study proposed that the genome of the mesophilic archaeon *Haloferax volcanii* was also largely packaged into nucleosomes (Ammar *et al.*, 2012). This study used micrococcal nuclease (MNase) sequencing, in which chromatin is digested with MNase at the nucleosome level and then subjected to deep sequencing. It remains to be determined whether the genome-wide protection of *H. volcanii* DNA from MNase digestion is attributed to histones or other non-histone NAPs (Hocher *et al.*, 2022).

**Fig. 2.**
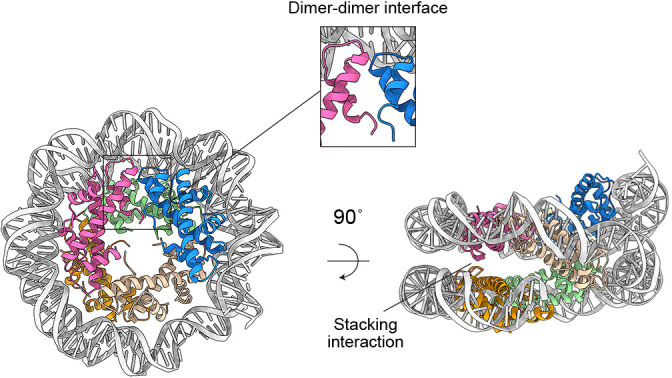
Structure of an archaeasome composed of the *Thermococcus kodakarensis* histone HtkA and 207 bp of Widom 601 DNA (EMPIAR: EMD-23403) (Bowerman *et al.*, 2021). HTkA dimers are indicated by different colors.

The emerging connection of thermophilic archaeal chromatin and eukaryotic chromatin is enticing, particularly since both contain histones as a major component. However, it is important to note that archaeal histones have distinctive features from those of eukaryotes. Archaeal histones form homodimers and heterodimers, whereas eukaryotic his­tones‍ ‍do not have the ability to form homodimers (Eickbush and Moudrianakis, 1978; Sandman *et al.*, 1994). Most archaeal histones lack N-terminal tails, whereas‍ ‍the N-terminal tails of eukaryotic histones play critical roles in‍ ‍altering local chromatin structure in response to signals (Henneman *et al.*, 2018; Ghoneim *et‍ ‍al.*,‍ ‍2021). Furthermore, the MNase digestion of chromatin isolated from three thermophilic archaea (*Methanothermobacter thermautotrophicus*, *T. kodakarensis*, and *Methanocaldococcus jannaschii*) indicates that archaeal histones assemble nucleosomes of varying sizes, ranging from 60 to ~500 bp in increments of ~30 bp. This property contrasts with the eukaryotic nucleosome, which consistently wraps ~147 bp of DNA (Luger *et al.*, 1997; Maruyama *et al.*, 2013; Nalabothula *et al.*, 2013; Ofer *et al.*, 2023). Structural ana­lyses of histones from two hyper­thermophilic archaea, *Methanothermus fervidus* and *T. kodakarensis*, revealed that the irregular size of archaeal nucleosomes originates from the unique ability of archaeal histone dimers to oligomerize through dimer-dimer interfaces and stacking interactions. This property results in the elongated superhelical winding of DNA (Fig. 2) (Mattiroli *et al.*, 2017; Bowerman *et al.*, 2021). The variable number of archaeal histone dimers that wrap a continuous DNA segment is referred to as “archaeasomes” or “hypernucleosomes” (Henneman *et al.*, 2018; Laursen *et al.*, 2021). Molecular dynamics simulations suggested that different archaeal histone paralogs possess varying capacities for oligomerization, raising the possibility that archaeal cells use specific histone paralogs to adjust the size of archaeasomes (Stevens *et al.*, 2020). Due to the absence of contrary evidence, it is assumed that this regulation of oligomerization occurs throughout the genome and does not target specific loci. This is in stark contrast to the more flexible regulation that eukaryotic chromatin achieves through histone tails (Stevens and Warnecke, 2023). While the archaeal mechanism of chromatin alteration appears to be less sophisticated, the development of extended histone oligomers and their global regulation may be advantageous for thermophilic archaea to protect their genomes from variable high temperatures.

## Emerging views on higher-order genome organization in thermophilic archaea

Genomic DNA is highly structured not only at a local scale, but also at larger scales, typically greater than 100‍ ‍kb. Since Walther Flemming first observed eukaryotic mitotic chromosomes, our knowledge of higher-order genome organization has steadily increased with the help of continuous technological advances. However, the focus of most investigations into the higher-order genome architecture has predominantly been on eukaryotes and bacteria. Therefore, the mechanisms by which archaeal genomes are folded on a large scale remain unclear.

In the 1990s and 2000s, Bernander and colleagues described the microscopic morphologies of fixed archaea with a focus on *Sulfolobus* cells. Their research revealed the non-random large-scale organization of archaeal genomes (Poplawski and Bernander, 1997; Malandrin *et al.*, 1999; Majerník *et al.*, 2005; Lundgren *et al.*, 2008). A decade later, a more detailed overview of the archaeal large-scale genome architecture was obtained by the application of chromosome conformation capture-based sequencing (Hi-C and 3C-seq) (Lieberman-Aiden *et al.*, 2009) to *Sulfolobus* archaea. The first Hi-C ana­lysis of *Sulfolobus* species revealed that the contact matrices of their genomes display a plaid-like interaction pattern (Takemata *et al.*, 2019), a hallmark of the existence of A/B compartments in eukaryotes (Lieberman-Aiden *et al.*, 2009). Similar to eukaryotic A/B compartments, the compartment structures of *Sulfolobus* genomes are differentiated by varying levels of gene expression, leading to the adoption of the A/B nomenclature. This finding was unexpected because A/B compartments had not previously been documented in prokaryotes. Subsequent higher-resolution 3C-seq ana­lyses revealed that *Sulfolobus* genomes are also folded into DNA loops and CID arrays (Takemata and Bell, 2021). The majority of DNA loops are anchored at operons encoding ribosomal subunits, and in many cases, these ribosomal genes interact with multiple other ribosomal loci (Takemata and Bell, 2021). *Sulfolobus* ribosomal genes may form a nucleolus-like hub structure that serves as a site for the transcription, translation, and assembly of ribosomal components.

How do *Sulfolobus* genomes establish their higher-order structures at the mole­cular level? Research revealed that *Sulfolobus* A/B compartments are formed by a unique *Sulfolobus*-specific protein named coalescin (ClsN), which is related to the SMC superfamily (Takemata *et al.*, 2019; Yoshinaga *et al.*, 2022). ClsN is distinguished by a conserved Cys-X-X-Cys motif at its center. While another SMC-like protein, Rad50, uses this motif for homodimerization, conventional SMC proteins instead use hinge domains for homo- or heterodimerization (Takemata and Bell, 2020). A subsequent phylogenetic study has positioned ClsN within a newly defined SMC clade, termed archaea-specific SMC-related proteins (ASRPs) (Yoshinaga *et al.*, 2022). ClsN preferentially binds to transcriptionally inactive loci and brings them into close proximity, thereby spatially segregating active and inactive genomic regions (Takemata *et al.*, 2019). Domain structures in *Sulfolobus* genomes, as the use of the term CID implies, are predominantly demarcated by high levels of transcription occurring at domain boundaries. ClsN also appears to assist the formation of CIDs within the B-type compartment (Takemata and Bell, 2021). The formation of ribosomal gene loops in *Sulfolobus* cells is also dependent on transcription (Takemata and Bell, 2021). Thus, transcription appears to underpin all of the different higher-order DNA structures in *Sulfolobus*. It is interesting to note that, despite the differing mole­cular mechanisms, the higher-order structures observed in *Sulfolobus* genomes are similar to those found in eukaryotes.

The biological role of the higher-order genome architecture in *Sulfolobus* remains less understood as compared to‍ ‍those of reverse gyrase and NAPs. An early study isolated‍ ‍a number of temperature-sensitive mutants of *S. acidocaldarius*, which displayed aberrant nucleoid morphologies and/or distributions upon the transition from 70°C to 81°C (Bernander *et al.*, 2000). The genes responsible for these phenotypes have not yet been identified, and how the altered genome architecture is linked to cell variability is unknown. A more recent study suggested that A/B compartments affect the evolution of *Sulfolobus* genomes. Analyses of various *Sulfolobus* species revealed that A-compartment genes are enriched in essential genes, exhibit a low density of single nucleotide polymorphisms, maintain well-conserved gene synteny, and display elevated levels of DNA accessibility. Conversely, genes in the B-type compartment exhibit the opposite traits. The authors suggested that the compartmentalization of *Sulfolobus* genomes restricts the access of B-compartment genes to repair machineries, differentiating the evolutionary rate of genes according to their compartmental identity (Badel *et al.*, 2022).

Hi-C/3C-seq ana­lyses were recently applied to two other thermophilic archaea (*T. kodakarensis* and *Thermofilum adornatum*) and two halophilic archaea (*H. volcanii* and *Halobacterium salinarum*), which demonstrated that different archaeal lineages use different modes of higher-order genome organization regardless of their habitats (Cockram *et al.*, 2021) (Sobolev* et al.*, 2021 3C-seq-captured chromosome conformation of the hyperthermophilic archaeon *Thermofilum* *adornatum*. bioRxiv. https://www.biorxiv.org/content/10.1101/2021.04.30.439615) (Table 1). All four species, along with members of the genus *Sulfolobus*, possess CIDs or CID-like structures. However, the A/B compartments appear to be unique to *Sulfolobus* species based on the presence/absence of a plaid-like contact pattern that correlates with gene expression. Discernible DNA loops have been detected in *Sulfolobus*, *H. volcanii*, and *H. salinarum*, but not in *T. adornatum*. It has not yet been established whether DNA loops are present or absent in *T. kodakarensis*. A caveat is that the apparent absence of loops in *T. adornatum* may be due to the insufficient resolution or read depth of Hi-C/3C-seq data. Although the underlying mole­cular mechanisms remain unclear, certain domain structures and DNA loops in *H. volcanii* are formed in a manner that is dependent on a canonical SMC protein. This dependence is reminiscent of eukaryotic TADs and loops. The rod-shaped archaeon *T. adornatum* is uniquely characterized by the juxtaposition of two halves of the circular genome. While many mesophilic rod-shaped bacteria use a canonical SMC complex to exhibit a similar genome arrangement (Le *et al.*, 2013; Lioy *et al.*, 2021), *T. adornatum* does not possess clear homologs of SMC complex subunits. *T. adornatum* instead possesses an SMC-like protein named Arcadin-4; however, its function is completely unknown (Ettema *et al.*, 2011). In conclusion, the higher-order organization of archaeal genomes is highly diverse at both the morphological and mechanistic levels, and these organizational variations do not appear to be associated with differences in growth temperature. The potential conformations of archaeal genomes may be restricted more by intrinsic factors (*e.g.*, cell shape, the arrangement of genes, and other functional elements on the genome) than by external environments. Archaea may have developed lineage-specific strategies to adapt their genome organization to these internal constraints within their respective habitats.

Do the variations in the higher-order architecture of archaeal genomes suggest that large-scale genome organization has a minimal impact on adaptability to high temperatures? It should be pointed out that Hi-C/3C-seq technologies cannot directly assess specific aspects of the 3D genome, such as the absolute chromosome volume. These structural features may be relevant to thermophilicity. Sabath and colleagues (Sabath *et al.*, 2013) indicated that a small chromosome volume is advantageous for adaptation to heat. Their findings revealed that genome size negatively correlates with habitat temperatures across a wide range of bacteria, including thermophiles. The authors suggested that the small genomes of thermophilic bacteria are the result of natural selection favoring a small cell size at high temperatures. Under thermally challenging conditions, cell size reductions may compensate for the energetic cost needed for non-growth-associated cellular maintenance (Sabath *et al.*, 2013). In addition to a decrease in the genome size, an elevated level of global chromosome compaction may contribute to cell volume reductions in thermophiles.

## Polyamines: mysterious compounds with potential roles in genome thermostability

Polyamines, positively charged aliphatic compounds, are ubiquitous across life forms. In contrast to other organisms, thermophilic prokaryotes synthesize a number of long-chain polyamines and branched polyamines in addition to common polyamines (Hamana *et al.*, 2003; Oshima, 2010). The cellular abundance of these unique polyamines increases with growth temperatures in the extreme thermophilic bacterium *Thermus thermophilus* and the hyperthermophilic archaeon *T. kodakarensis* (Morimoto *et al.*, 2010; Sakamoto *et al.*, 2022). The genetic disruption of pathways that produce long-chain or branched polyamines has been shown to reduce the cellular fitness of these organisms at high temperatures (Ohnuma *et al.*, 2005; Morimoto *et al.*, 2010; Sakamoto *et al.*, 2022). These findings suggest that certain polyamine species are critical for the survival of thermophiles.

Polyamines possibly contribute to the viability of thermophiles by interacting with DNA. The polycationic properties of polyamines are known to induce DNA compaction *in vitro* (Chattoraj *et al.*, 1978; Pelta *et al.*, 1996; Bloomfield, 1997; Higashibata *et al.*, 2000). A previous study using atomic force microscopy revealed that DNA forms a meshwork structure in the presence of a branched-chain polyamine, whereas the same DNA substrate adopts a more parallel arrangement in the presence of linear-chain polyamines (Muramatsu *et al.*, 2016). The meshwork structure may play a role in genome protection in response to heat shock. Polyamines also have a more local impact on DNA, such as converting the double helical structure of DNA from the B-form into the A-form, thereby increasing the melting temperature of DNA and preventing DNA from depurination (Hou *et al.*, 2001; Muramatsu *et al.*, 2016; Terui *et al.*, 2018). Long-chain and branched polyamines exert these effects more effectively than standard polyamines (Terui *et al.*, 2005, 2018; Muramatsu *et al.*, 2016).

## How do thermophilic bacteria and eukaryotes organize their genomes?

Most of the above discussion is based on findings obtained from thermophilic archaea, the most extensively studied thermophiles to date. How much of this knowledge is applicable to thermophiles outside the Archaea?

It currently remains unclear how thermophilic eukaryotes adapt their 3D genome structures to heat. While the upper temperature limit for bacteria and archaea is higher than 100°C, that for eukaryotes is approximately 60°C. The relatively low upper limit may be due to the low solubility of gasses in hot water because most eukaryotes heavily de­pend‍ ‍on oxygen for energy production (Rothschild and Mancinelli, 2001; Van Etten *et al.*, 2023). Thermophilic eukaryotes may have adapted to the modestly high temperature primarily by enhancing the thermostability of their proteins rather than evolving the specific organizational features of genomic DNA. It is also possible that, in the habitats of thermophilic eukaryotes, extensive arrays of nucleosomes are sufficient to protect genomic DNA (Weischet *et al.*, 1978).

Thermophilic bacteria have discernible features in common with thermophilic archaea, such as the use of reverse gyrase and the biogenesis of long-chain polyamines and branched polyamines (Oshima, 2010; Forterre, 2002). The hyperthermophilic bacterium *T. maritima* also appears to use the NAP HU to defend the genome against thermal damage (Mukherjee *et al.*, 2008). Although the 3D genome architecture in thermophilic bacteria remains under-investigated, at least some of the organizational strategies may be shared between thermophilic bacteria and archaea.

## Concluding remarks and future perspectives

The proper packaging of genomes for their storage, transmission, and use in transcription is a non-trivial challenge for all organisms. High environmental temperatures exacerbate this challenge, causing DNA denaturation, inducing chemical damage in DNA, and increasing chromosome fluctuations. *In vitro* studies revealed how reverse gyrase and NAPs potentially sculpt the organization of genomes and contribute to genome integrity in thermophilic archaea. Recent studies shed light on how histones and other NAPs have laid the foundation for the emergence of eukaryotic chromatin from thermophilic archaea. Chromosome conformation capture technologies have markedly expanded our knowledge of large-scale genome folding in thermophilic archaea. Despite these advances, it still remains elusive how thermophiles maintain genome integrity both at the structural and functional levels.

To obtain a more detailed understanding of genome organization in thermophiles, it is essential to clarify the *in vivo* mole­cular function of reverse gyrase because this enzyme is the most notable hallmark of extreme thermophiles and hyperthermophiles. Previous studies yielded conflicting results regarding whether reverse gyrase keeps intracellular DNA positively supercoiled and whether positive DNA supercoils contribute to the survival of thermophiles (Charbonnier and Forterre, 1994; Guipaud *et al.*, 1997; López-García and Forterre, 1999; López-García *et al.*, 2000; Couturier *et al.*, 2020; Villain *et al.*, 2021). However, an important caveat is that these studies investigated cellular DNA topology using a 2D-gel ana­lysis of extrachromosomal DNA. Therefore, the physiological significance of the reverse gyrase-mediated introduction of positive DNA supercoils on the genome remains unclear. To address this issue, genome-wide mapping of supercoiled DNA employing tools such as psoralen (a binder of negative DNA supercoils) and GapR (a binder of positive DNA supercoils) will be needed (Bermúdez *et al.*, 2010; Naughton *et al.*, 2013; Guo *et al.*, 2021). Furthermore, it will be imperative to elucidate what kind of DNA damage is induced by high temperatures in reverse gyrase mutants and how this relates to the loss of positive DNA supercoils.

*In vivo* characterization is also highly demanded for a more detailed understanding of NAP functions in thermophiles. Reverse genetics and transposon mutagenesis revealed that certain NAPs, individually or in combination, are essential for the viability of thermophilic archaea (Čuboňvá *et al.*, 2012; Zhang *et al.*, 2018). CRISPR-mediated RNA degradation could be a potential approach to conditionally inactivate the essential functions mediated by these NAPs. However, this method needs to be further improved for quick and efficient protein depletion (Peng *et al.*, 2015; Zebec *et al.*, 2014). Future studies are also required to delineate the distribution of different NAPs across the genomes of thermophiles. Of particular interest are where archaeasomes are positioned and how their locations and sizes are affected by different histone paralogs, given their potential linkage to eukaryotic chromatin.

Research in the last five years has successfully outlined the higher-order genome organization in archaea (Bell, 2022). However, due to the emerging organizational diversity of archaeal genomes, it remains unclear how a large-scale genome architecture impacts the biology of thermophilic archaea. Additional Hi-C/3C-seq studies across various archaeal lineages will aid in resolving this issue. Although not yet examined, it is possible that reverse gyrase and NAPs regulate the higher-order genome architecture in thermophilic archaea. Further studies are needed to clarify how this regulation occurs as well as its contribution to genome integrity at high temperatures. Furthermore, the mechanisms by which large-scale genome organization coordinates with cell cycle progression need to be examined in detail because it is a topic that is currently underexplored in both thermophilic and mesophilic archaea. *S. acidocaldarius* will be an excellent model to address this knowledge gap given the availability of cell cycle synchronization methods and live-cell DNA imaging technology for this organism (Duggin *et al.*, 2008; Pulschen *et al.*, 2020; Risa *et al.*, 2020). This research direction will be further fostered by combining live-cell imaging and recently developed thermostable fluorescent proteins that work even at 80°C (Campbell *et al.*, 2022).

The *in vivo* role of polyamines in shaping the 3D genomes of thermophiles is largely unknown. Addressing this question requires a more detailed understanding of polyamine metabolism and its genetic manipulation. However, the elucidation of polyamine biogenesis is challenging due to the difficulties associated with detecting and quantifying various polyamines. Future improvements in analytical methods, such as liquid chromatography, will help to‍ ‍overcome this issue. Once a more comprehensive understanding of polyamine metabolism is achieved, the *in vivo* function of a specific polyamine can be tested by genetically depleting the compound and investigating its impact by Hi-C/3C-seq and other technologies.

There is extremely limited knowledge on how the different aspects of genome organization described in this review are interlinked in thermophiles. For example, it remains to be determined how the DNA supercoiling activity of reverse gyrase affects genome architecture at the scale of plectoneme structures (typically ~10 kb in bacteria) (Le *et al.*, 2013) and beyond. *In vitro* studies suggest that NAPs not only affect local DNA structures, but also mediate longer-range DNA interactions by constraining DNA supercoils or bridging DNA segments (Guo *et al.*, 2008; Hardy and Martin, 2008; Laurens *et al.*, 2012; Ofer *et al.*, 2023). However, this possibility needs to be tested *in vivo*. By exploring the interplay among various facets of the 3D genome, we will be able to answer the question of how thermophiles organize their genomes.

## Citation

Takemata, N. (2024) How Do Thermophiles Organize Their Genomes?. *Microbes Environ ***39**: ME23087.

https://doi.org/10.1264/jsme2.ME23087

## Figures and Tables

**Table 1.** Large-scale genome structures and potential genome organizers in different archaeal species

Organism (phylum)	Optimal growth temperature	Topoisomerases^1^	A/B compart-ment^2^	Domains^2^	Loops^2^	Other features^2^	NAPs^3^	SMC and SMC-related proteins^4^
*Sulfolobus acidocaldarius* (Crenarchaeota)	75–78°C	Topo III, Topo VI, reverse gyrase	Yes	Yes	Yes	NA	Alba, Sul7, Cren7	ClsN
*Thermofilum adornatum* (Crenarchaeota)	80°C	Topo III, Topo VI, reverse gyrase	No	Yes	No	Juxtaposition of two chromosome halves	Alba, histone	Arcadin-4
*Thermococcus kodakarensis* (Euryarchaeota)	85°C	Topo III, Topo VI, reverse gyrase	No	Undeter-mined	Undeter-mined	NA	Alba, histone, TrmBL2	Smc
*Haloferax volcanii* (Euryarchaeota)	45°C	Topo III, Topo VI, gyrase	No	Yes	Yes	NA	Histone, MC1	Smc, Sph
*Halobacterium salinarum* (Euryarchaeota)	37°C	Topo III, Topo VI, gyrase	No	Yes	Yes	NA	Histone, MC1	Smc, Sph

^1^ See Garnier *et al.*, 2021. ^2^ See Takemata *et al.*, 2019; Cockram *et al.*, 2021; Takemata and Bell, 2021; Sobolev *et al.*, 2021 3C-seq-captured chromosome conformation of the hyperthermophilic archaeon *Thermofilum adornatum*. bioRxiv. https://www.biorxiv.org/content/10.1101/2021.04.30.439615. ^3^ See Peeters *et al.*, 2015; Hocher *et al.*, 2022. ^4^ See Yoshinaga *et al.*, 2022.
